# Integrated Characterization of AP-2δ Reveals Distinct Regulatory Architecture in Lung Adenocarcinoma and Lung Squamous Cell Carcinoma

**DOI:** 10.3390/cancers18081278

**Published:** 2026-04-17

**Authors:** Damian Kołat, Weronika Kruczkowska, Żaneta Kałuzińska-Kołat, Cromwel Tepap Zemnou, Mateusz Kciuk, Lin-Yong Zhao, Renata Kontek, Elżbieta Płuciennik

**Affiliations:** 1 Department of Functional Genomics, Medical University of Lodz, 90-752 Lodz, Poland; weronika.kruczkowska@umed.lodz.pl (W.K.); zaneta.kaluzinska@umed.lodz.pl (Ż.K.-K.); mateusz.kciuk@biol.uni.lodz.pl (M.K.); elzbieta.pluciennik@umed.lodz.pl (E.P.); 2 Department of Molecular Biotechnology and Genetics, University of Lodz, 90-237 Lodz, Poland; renata.kontek@biol.uni.lodz.pl; 3 International Doctoral School, Medical University of Lodz, Hallera Square 1, 90-647 Lodz, Poland; 4 School of Engineering BioMedTech, EuroMed University of Fes (UEMF), Fes 30070, Morocco; c.tepapzemnou@ueuromed.org; 5 Department of General Surgery, West China Hospital, Sichuan University, Chengdu 610041, China; 153795352@scu.edu.cn

**Keywords:** AP-2δ, TFAP2D, lung adenocarcinoma, lung squamous cell carcinoma, bioinformatics, expression patterns, clinical profiling, chromatin compartments, differential coexpression, ligandability

## Abstract

**Background/Objectives**: AP-2δ, encoded by *TFAP2D*, is one of the least characterized members of the AP-2 transcription factor family, although available evidence suggests biologically relevant roles in lung cancer that have not yet been thoroughly examined. The aim of the present study was to provide an integrated characterization of AP-2δ/*TFAP2D* in lung adenocarcinoma (LUAD) and lung squamous cell carcinoma (LUSC). **Methods**: LUAD and LUSC data were obtained from The Cancer Genome Atlas. The analysis comprised the expression profiling of AP-2δ target genes, survival-guided *TFAP2D* stratification, clinical profiling, differential expression and intersection analyses, methylation-derived chromatin compartment profiling, *TFAP2D*-associated cofactor rewiring, and genome-wide enrichment of AP-2δ targets. In parallel, pocket prioritization was performed using an AlphaFold model of AP-2δ with cross-tool consensus mapping. **Results**: *TFAP2D* stratification delineated biologically-distinct states in both histological subtypes (LUAD and LUSC). AP-2δ target genes showed subtype-specific expression patterns and functional organization. The consistent survival association was observed for progression-free interval rather than uniformly across all endpoints. Clinical profiling was more closely associated with molecular subtype composition than broad clinicopathological differences. Differential expression analyses identified both shared and histology-dependent programs associated with *TFAP2D*. In the chromatin-compartment analysis, LUSC showed a broader and more coherent footprint, whereas LUAD displayed more selective cofactor rewiring. Structure-based analysis prioritized a small set of reproducible candidate pockets concentrated within ordered regions of the TF_AP-2 domain. **Conclusions**: AP-2δ marks biologically meaningful but histologically non-uniform regulatory states in lung cancer. These findings provide an integrated framework for understanding *TFAP2D*-dependent regulation in LUAD and LUSC, highlighting AP-2δ as a candidate for future mechanistic and translational investigation.

## Simple Summary

AP-2δ, encoded by *TFAP2D*, is one of the least characterized members of the AP-2 transcription factor family, although available evidence suggests biologically relevant roles in lung cancer. This study investigates the role of AP-2δ/*TFAP2D* in lung adenocarcinoma (LUAD) and lung squamous cell carcinoma (LUSC) using a computational workflow investigating expression patterns, survival-linked stratification, clinical profiling, differential expression, chromatin context, cofactor coupling, and structural tractability. *TFAP2D* stratification delineated biologically distinct states in both histological subtypes, with differing associated regulatory patterns: LUSC showed a broader chromatin-compartment footprint, whereas LUAD displayed more selective cofactor rewiring. Additionally, structure-based analysis identified a small set of reproducible candidate pockets in AP-2δ. These findings provide an integrated framework for understanding *TFAP2D*-dependent regulation in LUAD and LUSC, highlighting AP-2δ as a candidate for future investigation in translational cancer research.

## 1. Introduction

The activating enhancer-binding protein-2 (AP-2) family comprises five protein-coding members (AP-2α, AP-2β, AP-2γ, AP-2δ, and AP-2ε) encoded by the *TFAP2A*, *TFAP2B*, *TFAP2C*, *TFAP2D*, and *TFAP2E* genes, respectively. These transcription factors (TFs) maintain high sequence conservation across species and serve as crucial regulators during embryogenesis and cancer development [1]. Each AP-2 protein shares a similar domain architecture, with an N-terminal transactivation domain and a conserved C-terminal module comprising a basic DNA-binding region followed by a helix-span-helix domain that supports the homo- and hetero-dimerization required for sequence-specific DNA binding. Through these features, AP-2 TFs coordinate diverse cellular programs, e.g., proliferation, differentiation, apoptosis, and epithelial–mesenchymal plasticity, supporting their broad relevance to tissue morphogenesis and neoplastic progression [2,3]. While AP-2α, AP-2β, and AP-2γ have been extensively characterized since their initial discovery, relatively limited investigation has been conducted on AP-2δ and AP-2ε, particularly their roles in cancer [4,5,6]. Among them, AP-2δ has been examined in relatively few studies, although existing reports suggest that it may be relevant in specific tumors. This imbalance may be partially attributed to its more tissue-restricted developmental expression pattern relative to other AP-2 family members, which has likely limited functional exploration in cancer settings [2,7].

AP-2δ exhibits several distinctive structural and functional characteristics that set it apart from other AP-2 family members. For instance, while AP-2α, AP-2β, AP-2γ, and AP-2ε contain a conserved proline-rich motif in their transactivation domains, AP-2δ features a histidine-rich region while also retaining the architecture of the amino- and carboxy-terminal ends. In addition, AP-2δ exhibits markedly lower binding affinity to conserved AP-2-binding sites than other family members [8,9,10]. These differences have prompted the view that AP-2δ may operate through distinct transactivation mechanisms, potentially via a different cofactor repertoire and by targeting alternative DNA-binding sites, or even act as a negative regulator that modulates the activity or DNA-binding capacity of other AP-2 factors [4,11]. Importantly, its elevated number of post-translational modification sites, extended β-strand regions, and fold features consistent with potential ligandability suggest that AP-2δ may be one of the more structurally tractable family members [8]. Moreover, it is also possible that AP-2δ influences chromatin organization [8]. Therefore, given the limited characterization to date, its distinctive features, and predicted ligandability, AP-2δ appears as an intriguing candidate for focused investigation in cancer contexts.

One such area meriting particular study is lung cancer. It remains the leading cause of cancer-related mortality worldwide, with non-small cell lung cancer (NSCLC) accounting for approximately 85% of all cases. In NSCLC, lung adenocarcinoma (LUAD) and lung squamous cell carcinoma (LUSC) are the dominant histological subtypes, together accounting for over 70% of NSCLC diagnoses [12,13,14]. LUAD and LUSC differ substantially in mutational landscapes, gene expression profiles, and clinical behavior, reinforcing the need for subtype-aware analyses and therapeutic strategies [15,16]. Despite advances in targeted and immune-based therapies, the five-year survival rate for lung cancer remains low, underscoring the need to clarify underexplored regulatory mechanisms and identify additional actionable vulnerabilities [17,18]. In this context, *TFAP2D* mutations have been reported across multiple cancers, including LUAD and LUSC, yet the functional implications of these alterations remain largely unexplored [19].

The structural characteristics and ligandability of the five AP-2 family members have been detailed in previous research integrating data from multiple repositories [8]. Among the family members, AP-2δ was identified as the most informative candidate for further structural investigation. The study then utilized preliminary, tumor-oriented analyses to prioritize cancer types for a deeper AP-2δ study; the results highlighted lung cancer (particularly LUAD and LUSC) based on *TFAP2D* expression patterns and other indicators of potentially functional relevance. *TFAP2D* expression was found to differ between LUAD and LUSC, supporting the possibility that AP-2δ may play subtype-specific roles in lung cancer. However, the study was intentionally designed to compare protein-coding AP-2 family members rather than to provide an in-depth characterization of AP-2δ in a specific tumor context. Consequently, lung cancer was addressed only in a focused section, and no detailed analyses investigating AP-2δ-associated transcriptional programs were performed. A deeper understanding is therefore needed of how *TFAP2D* expression relates to subtype-specific transcriptomic patterns and how these relationships align with the distinct biological features of LUAD and LUSC.

Thus, the present study was designed to provide an integrated characterization of AP-2δ/*TFAP2D* in lung adenocarcinoma and lung squamous cell carcinoma. The workflow combined expression profiling with survival-linked stratification, clinical and molecular subclassification analyses, cross-cohort differential expression analysis, methylation-derived chromatin compartment analysis, cofactor rewiring, and structure-guided pocket prioritization. The resulting framework indicated that *TFAP2D* stratification delineates biologically distinct states in both LUAD and LUSC, and is associated with progression-free interval and molecular subclassification. *TFAP2D* stratification further revealed that the associated regulatory architecture is not identical, with broader shifts in chromatin compartment organization noted in LUSC, and more pronounced selective cofactor rewiring in LUAD. The analysis also enabled prioritizing a small set of structurally credible candidate pockets, with the leading candidates localized within the conserved TF_AP-2 domain. Collectively, these findings provide a foundation for future studies on *TFAP2D*-centered regulatory mechanisms, prognostic relevance, and histology-specific vulnerabilities in lung cancer.

## 2. Materials and Methods

### 2.1. Acquisition of LUAD/LUSC Datasets and AP-2δ Target Genes

Transcriptomic data for LUAD and LUSC were obtained from the Genomic Data Commons (GDC) repository [20] (accessed on 28 October 2025) using the GDCquery() function in the TCGAbiolinks v2.34.1 R-package (R Foundation for Statistical Computing, Vienna, Austria). Tumor data were downloaded as raw counts generated with the Spliced Transcripts Alignment to a Reference (STAR) protocol, with the hg38 build used as the reference genome. Data were additionally retrieved as normalized values (transcripts per million; TPM), which were used for expression profiling and survival analysis, whereas raw counts were retained for differential expression analysis. Clinical profiles were assembled from GDC and the TCGA Clinical Data Resource (TCGA-CDR) [21], the latter serving as the source of clinical endpoints, *viz.* overall survival (OS), disease-specific survival (DSS), disease-free interval (DFI), and progression-free interval (PFI). In addition, Illumina HumanMethylation450 (450k) tumor data for LUAD and LUSC were retrieved from TCGA for chromatin compartment analyses (more details in Section 2.7). A list of genes targeted by AP-2δ was obtained from our previous research [8,22].

### 2.2. Evaluation of Expression Patterns, Detection of Gene Modules, and Their Functional Annotation

The expression dynamics of AP-2δ target genes were evaluated across LUAD and LUSC using the Monocle3 toolkit [23] (University of Washington, Seattle, WA, USA). Genes with no detectable expression in the analyzed samples were excluded before subsequent steps. During pre-processing *via* preprocess_cds(), the num_dim parameter was set to 100. Dimensionality reduction was executed with reduce_dimension(), and clustering was carried out with cluster_cells(); both steps used Uniform Manifold Approximation and Projection (UMAP) as the reduction_method for baseline clustering. Statistical validation was performed using Moran’s I spatial autocorrelation in graph_test() with neighbor_graph = “knn” and a q-value cutoff of 0.05. Genes were further partitioned into coexpression modules using the Louvain community detection approach via find_gene_modules(), with default parameters (umap.min_dist = 0.1, umap.n_neighbors = 15, k = 20). Module patterns were summarized using Ward.D2 hierarchical clustering and visualized with pheatmap(). All steps followed the official Monocle3 documentation (https://cole-trapnell-lab.github.io/monocle3, accessed on 13 November 2025).

Because the first module contained substantially more genes than the remaining modules, feature ranking was applied to reduce the influence of module size on functional interpretation. For this purpose, Multiple Support Vector Machine Recursive Feature Elimination (mSVM-RFE) [24] was used. Feature/gene ranking was carried out with svmRFE() using 10-fold cross-validation (k = 10) and halve.above = 100, with the e1071 R-package (R Foundation for Statistical Computing, Vienna, Austria) loaded to enable SVM fitting. After ranking features across all training folds, consensus feature lists were generated with WriteFeatures() and ordered by ascending AvgRank (lower values indicating higher priority). The top 50 genes from the first module were then carried forward for functional annotation, along with the complete gene sets from the other modules. Downstream analysis was performed in Metascape [25] using the “Multiple Gene Lists” and “Express Analysis” options. Pathway and process enrichment was evaluated against the whole-genome background using Metascape’s built-in ontological resources; terms with *p* < 0.01 and enrichment factor >1.5 were retained and grouped into clusters based on membership similarity.

### 2.3. Survival Analysis

Optimal *TFAP2D* expression thresholds for stratifying patients with LUAD and LUSC based on OS, DSS, DFI, and PFI were identified using the EvaluateCutpoints tool (Medical University of Lodz, Lodz, Poland) [26] with the “Cutp” method for cutoff determination. Kaplan–Meier curves were obtained with the survfit() function (survival v3.8-3 R-package) and visualized with the ggsurvplot() function (survminer v0.5.0 R-package) and hazard ratios (HRs) were estimated with the coxph() function from the survival v3.8-3 R-package (R Foundation for Statistical Computing, Vienna, Austria), whereas forest plots were constructed using the forest_model() function from the forestmodel v0.6.2 R-package (R Foundation for Statistical Computing, Vienna, Austria). The proportional hazards assumption of Cox regression models was assessed using Schoenfeld residuals with the cox.zph() function (survival v3.8-3 R-package) and ggcoxdiagnostics() function (survminer v0.5.0 R-package; R Foundation for Statistical Computing, Vienna, Austria). Given the results of the survival analysis (see Section 3.2), for further investigation, the LUAD/LUSC cohorts were dichotomized according to the PFI into “*TFAP2D*-high” and “*TFAP2D*-low” groups, henceforth denoting patients with higher and lower *TFAP2D* expression within each cohort.

### 2.4. Clinical Profiling

The *TFAP2D*-stratified groups within the LUAD and LUSC cohorts were compared with regard to the clinical data. Continuous variables were evaluated for within-group normality *via* the Shapiro–Wilk test. Those with a normal distribution were compared using Welch’s *t*-test, and those without using the Wilcoxon rank-sum test. Effect sizes were reported as Hedges’ g (*t*-tests) or rank-biserial correlation (Wilcoxon), with 95% confidence intervals (CIs) computed using the effectsize v1.0.1 R-package (R Foundation for Statistical Computing, Vienna, Austria). Categorical variables were tested using Pearson’s χ^2^; when contingency tables were sparse, χ^2^ *p*-values were estimated by Monte-Carlo simulation (10,000 replicates), and effect sizes were summarized using Cramér’s V with confidence intervals. For categorical variables showing evidence of a global group difference, Fisher’s exact test was used to identify the categories driving the association, with effect sizes reported as the odds ratios (ORs) and corresponding confidence intervals. Multiple testing across clinical variables was controlled within each cohort using the Benjamini–Hochberg (BH) false discovery rate (FDR), and the results were visualized with the ggstatsplot v0.13.4 R-package (R Foundation for Statistical Computing, Vienna, Austria).

### 2.5. Differential Expression Analysis

For LUAD and LUSC individually, the limma-voom method (limma v3.62.2 R package; R Foundation for Statistical Computing, Vienna, Austria) [27,28] was applied to identify genes differentially expressed between patient groups with higher and lower *TFAP2D* expression. The pipeline involved Trimmed Mean of M-values (TMM) normalization *via* calcNormFactors() and filtering to retain genes with ≥5 counts per million in at least one library. Variance was modeled using the voom() transformation, and a cohort-specific design matrix was fitted with lmFit() using weighted least squares to compare the *TFAP2D*-high and *TFAP2D*-low groups. To calculate log_2_ fold-change values (log2FC), contrasts were specified with makeContrasts(), followed by contrasts.fit() and empirical Bayes moderation of standard errors with eBayes(). Differentially expressed genes were retrieved with topTable() using Benjamini–Hochberg FDR correction, with thresholds of |log_2_FC| > 0.6 and adjusted *p*-value < 0.05.

### 2.6. Intersection Analysis and Gene Ontology

The genes differentially expressed between *TFAP2D*-high and *TFAP2D*-low groups for LUAD were intersected with those from the analogous LUSC comparison. The resulting overlap was visualized as a Venn diagram using the draw.pairwise.venn() function from the VennDiagram v1.7.3 R-package (R Foundation for Statistical Computing, Vienna, Austria). The gene sets derived from the intersection were functionally annotated in DAVID [29] using default parameters, with the official gene symbol as the input identifier. The analysis was restricted to GO biological processes, KEGG, and Reactome resources, and the top three most significant terms per ontological category were retained. To visualize the gene sets common to the *TFAP2D* strata in LUAD and LUSC, heatmaps were generated using the ComplexHeatmap v2.27.1 R-package (R Foundation for Statistical Computing, Vienna, Austria). The samples were arranged into cohort and *TFAP2D* strata and hierarchically clustered within each stratum (correlation distance; average linkage). The genes were independently split into predefined categories and hierarchically clustered within each category using the same distance metric and linkage method.

### 2.7. Chromatin Compartment Profiling and Stratified Comparisons

The TCGA Illumina HumanMethylation450 data for LUAD and LUSC were mapped to hg38, and probes were aggregated into non-overlapping 100 kb genomic bins. Genomic interval handling and bin-level aggregation were performed using the GenomicRanges v1.58.0 R-package (R Foundation for Statistical Computing, Vienna, Austria), with reference-track import and coordinate harmonization handled via standard genome-annotation workflows. To ensure consistent A/B compartment sign interpretation across cohorts (with A denoting the open/active chromatin state and B the closed/inactive state), a published TCGA-based 100 kb compartment eigenvector reference track was used as an external orientation baseline [30]. Analyses were restricted in both cohorts to bins covered by this reference, and the sign convention was aligned so that one sign corresponded to the A/open state and the opposite sign to the B/closed state. Per-sample A/B calls were derived for each retained 100 kb bin and summarized within *TFAP2D*-high and *TFAP2D*-low strata as the fraction of samples assigned to the open/A-like state, with the B/closed fraction treated as the complementary proportion; the between-group difference was denoted as Δp_open.

Bin-wise group differences between *TFAP2D*-high and *TFAP2D*-low samples were evaluated using standard two-sample tests implemented in the stats v4.4.3 R-package (R Foundation for Statistical Computing, Vienna, Austria), followed by Benjamini–Hochberg FDR correction across bins. Significant bins were then merged into contiguous genomic blocks using range-reduction in the GenomicRanges v1.58.0 R-package (R Foundation for Statistical Computing, Vienna, Austria), requiring at least three consecutive significant 100-kb bins. For downstream interpretation, functional overlap was assessed using Fisher’s exact test separately for individual significant bins (SigBins) and merged significant blocks (SigBlocks). Descriptive summaries of bin- and block-level distributions were reported as the median and interquartile range (IQR). Summary enrichment matrices were visualized using the ComplexHeatmap v2.27.1 R-package (R Foundation for Statistical Computing, Vienna, Austria), whereas directional genome-wide summaries were generated as Manhattan plots in ggplot2 v4.0.1 R-package (R Foundation for Statistical Computing, Vienna, Austria).

### 2.8. TFAP2D-Associated Cofactor Rewiring and Chromatin-Context Integration

Gene coexpression related to *TFAP2D* was assessed separately in LUAD and LUSC using TMM-normalized expression matrices generated with the edgeR v4.4.2 R-package (R Foundation for Statistical Computing, Vienna, Austria). Within each cohort, Pearson correlations were computed between *TFAP2D* expression and each gene using standard functions from the stats v4.4.3 R-package (R Foundation for Statistical Computing, Vienna, Austria); genes were ranked by absolute correlation coefficient (|r|). A panel of transcriptional cofactors (TcoFs) of AP-2δ [8] was then evaluated within each cohort by differential coexpression analysis, hereafter referred to as cofactor rewiring. Pearson correlations r(*TFAP2D*, TcoF) were calculated separately in *TFAP2D*-high and *TFAP2D*-low strata, and their difference (hereafter denoted as Δr) was tested using Fisher’s r-to-z transformation. Multiple testing across the TcoFs was controlled by Benjamini–Hochberg FDR.

Chromatin-context integration was performed using the same methylation-derived A/B compartment framework described in Section 2.7. For each cohort, compartment composition was summarized for the *TFAP2D*-associated top 500 ranked neighborhoods and contrasted using Fisher’s exact tests on A/B proportions implemented in the stats v4.4.3 R-package (R Foundation for Statistical Computing, Vienna, Austria). Finally, enrichment of AP-2δ targets [8,22] was assessed genome-wide using a preranked running-enrichment approach implemented in the fgsea v1.32.4 R-package (R Foundation for Statistical Computing, Vienna, Austria), applied to the full gene ranking by |r|. Significance was estimated by permutation and summarized as enrichment scores and nominal *p*-values. Plots were generated with the ggplot2 v4.0.1 R-package (R Foundation for Statistical Computing, Vienna, Austria).

### 2.9. Structure-Based Pocket Identification and Unified Prioritization

A full-length AlphaFold model of AP-2δ (AF-Q7Z6R9-F1-model_v6.pdb, 452 aa) was used as the structural substrate for ligandability assessment. Residue-level model confidence was derived from the predicted local distance difference test (pLDDT); the result was used for quality control and residue-level pocket contextualization. Candidate cavities were evaluated with four complementary tools capturing distinct evidence layers: PrankWeb/P2Rank [31] for machine learning-based pocket detection and pocket-level confidence descriptors, FTMap [32] for fragment hotspot mapping, MOLE 2.5 [33] for tunnel connectivity and bottleneck metrics, as well as DoGSite3 [34] for geometry-based cavity evaluation and descriptors such as pocket depth, volume, enclosure, and surface/volume relationships. Tool outputs were reconciled by spatial and residue-level overlap, and cross-tool consensus candidates were retained as unified pockets (UPs). Additionally, R1-like metrics were calculated from the complete DoGSite3 ranking. To determine the mutational context, data from cBioPortal (Memorial Sloan Kettering Cancer Center, New York, NY, USA) [35] for TCGA-LUAD and TCGA-LUSC were converted to residue coordinates and aggregated as mutated-sample counts per residue. These positions were aligned to the pLDDT axis and overlaid with UP residue tracks to enable residue-level comparison between model confidence and pocket location. The structure of the unified pockets was inspected using ChimeraX v1.11.1 (University of California, San Francisco, CA, USA) [36].

## 3. Results and Discussion

### 3.1. AP-2δ Target Genes Exhibit Subtype-Specific Expression Patterns and Modular Functional Organization in Lung Cancer

**Figure 1:**
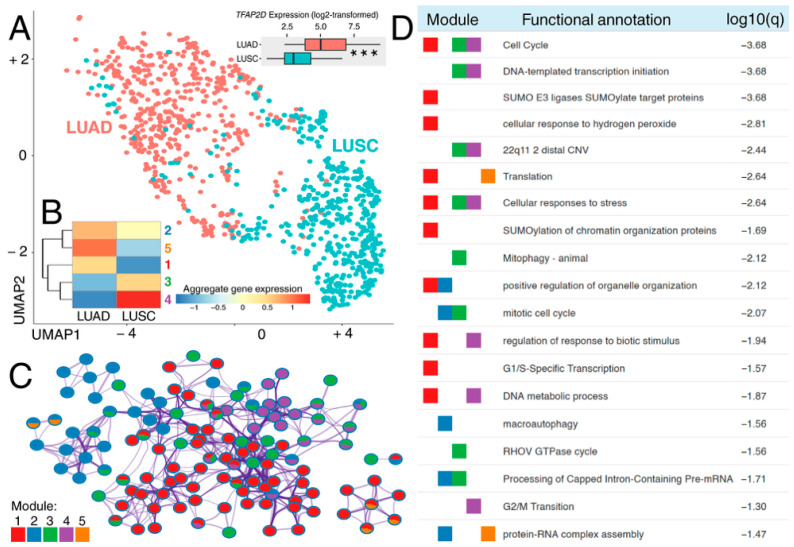
Transcriptomic and functional profiling of AP-2δ target genes in LUAD and LUSC. (**A**) UMAP projection of LUAD and LUSC samples based on the gene expression of AP-2δ targets, with accompanying comparison of *TFAP2D* expression between cohorts. (**B**) Aggregate expression patterns of five gene modules identified during transcriptomic profiling. (**C**) Functional enrichment network derived from module-wise analysis. (**D**) Representative enriched biological categories for gene modules. *p* < 0.001 (***).

Expression profiling of AP-2δ target genes separated the LUAD from LUSC samples, indicating that the associated transcriptomic landscape differs substantially between these two major NSCLC subtypes (Figure 1A). Moreover, *TFAP2D* was more highly expressed in LUAD than in LUSC (*p* < 0.001), suggesting a histology-dependent role. Module-level decomposition resolved five gene clusters, with the expression of genes from modules 1 and 5 shifted toward LUAD and those of modules 3 and 4 toward LUSC (Figure 1B). Hence, when understanding the biological aspect of this variation, it is important to view it in terms of modular organization rather than single-gene differences. This broader architecture is consistent with the behavior of AP-2 family regulators, whose output depends not only on DNA binding but also on chromatin accessibility and interacting cofactors. However, the evidence for such context dependence comes mainly from other AP-2 paralogs rather than AP-2δ itself. CITED4 has been reported to interact with most AP-2 isoforms and to coactivate them in a cell type- and isoform-dependent manner, whereas AP-2α can be coactivated by p300/CBP in the presence of CITED2, thus supporting a family-level model of cofactor-sensitive transcriptional control [37,38,39].

Network analysis showed that these modules were not biologically isolated, but converged on an interconnected set of enriched processes (Figure 1C). This architecture suggests that *TFAP2D*-linked programs in LUAD and LUSC are organized around partially overlapping regulatory neighborhoods. Such a pattern is compatible with context-dependent rewiring rather than a single conserved downstream program: different modules may still converge on related biological processes, but through distinct transcriptional components. This interpretation also aligns with the broader perspective of the AP-2 family, in which chromatin engagement and enhancer activity vary across paralogs and cellular states rather than being fixed properties of the whole family [4,39].

Functional annotation of the five modules identified a broad spectrum of cancer-relevant biological processes (Figure 1D). The dominant enrichment signal was cell cycle regulation (log10(q) = −3.68), with G_1_/S-specific transcription (log10(q) = −1.57) and G_2_/M transition (log10(q) = −1.30) also represented; these results position the targets of AP-2δ within major cell cycle stages. Other enriched categories included cellular response to stress, SUMOylation of chromatin organization proteins, RNA-processing functions, metabolic processes, and cellular response to hydrogen peroxide. Dysregulation of cell cycle checkpoints is a recognized feature of carcinogenesis [40], and altered RNA splicing is a therapeutically relevant layer of lung cancer biology [41]. The SUMO-related signal is also notable, as SUMO pathway components have been linked to NSCLC biology through both tumor-promoting and tumor-restraining mechanisms, including SUMO1-dependent proliferation and invasion, as well as PIASy-mediated antagonism of RAS-driven survival [42,43]. The oxidative stress-related terms are also consistent with the established role of redox imbalance in pulmonary carcinogenesis [44]. Overall, the network structure suggests that genes with different expression patterns in LUAD and LUSC can converge on the same biological processes, implying context-dependent rather than unidirectional *TFAP2D*-associated regulation. This places AP-2δ within a broader regulatory architecture spanning proliferation, stress response, RNA processing, and post-translational control rather than a single isolated pathway.

### 3.2. TFAP2D Expression Shows a Consistent Prognostic Association with Progression-Free Interval

**Figure 2:**
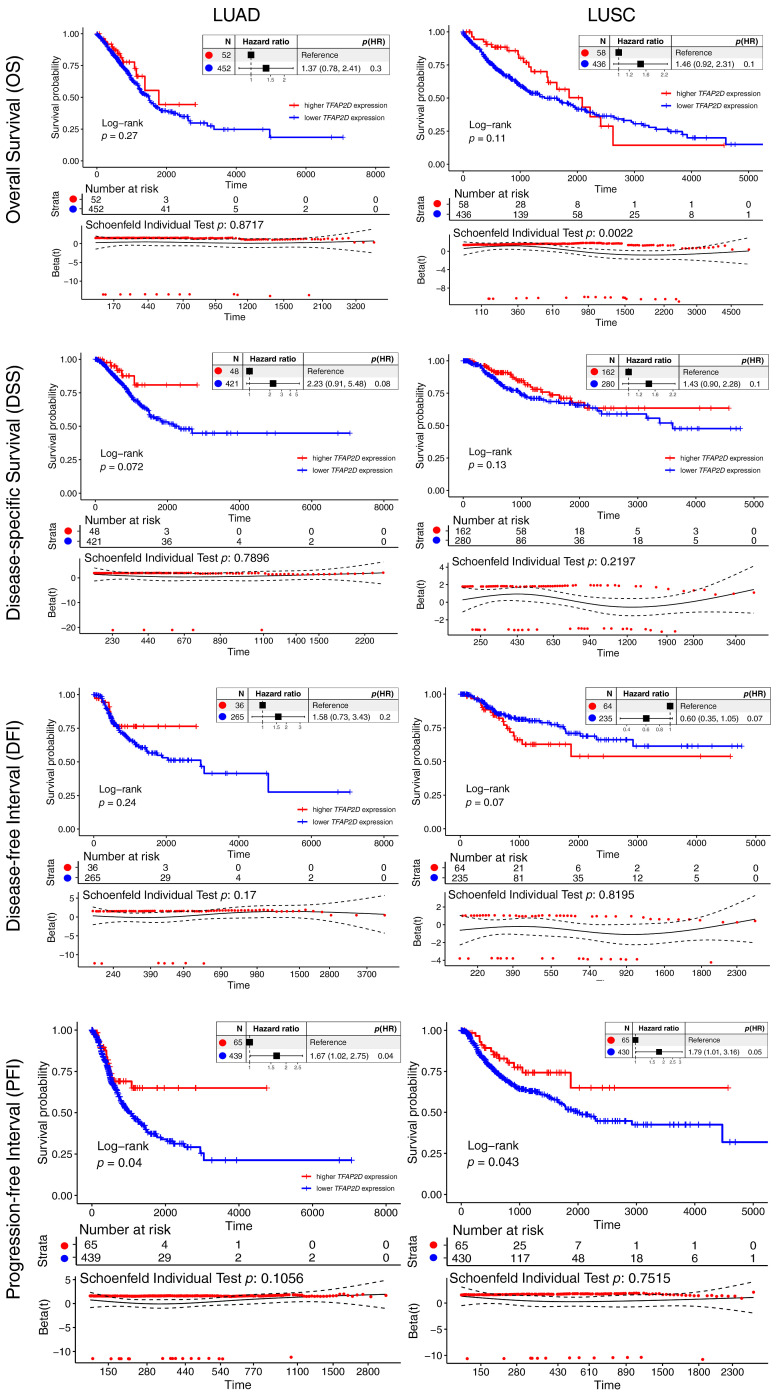
Survival analysis of *TFAP2D*-stratified LUAD and LUSC cohorts. Rows correspond to individual clinical endpoints (OS, DSS, DFI, PFI), with LUAD and LUSC shown side by side. Each panel includes the number of patients at risk at specified time points, hazard ratios with 95% confidence intervals, log-rank *p*-values, and proportional hazard diagnostics.

Survival analysis revealed a non-uniform prognostic signal associated with *TFAP2D* across the four clinical endpoints (Figure 2). Neither OS, DSS, nor DFI differed significantly between *TFAP2D* strata in either LUAD or LUSC. Instead, a consistent association was noted for PFI: the *TFAP2D*-low group showed a significantly shorter progression-free interval than the *TFAP2D*-high group in LUAD (HR = 1.67, 95% CI: 1.02–2.75; log-rank *p* = 0.04) and in LUSC (HR = 1.79, 95% CI: 1.01–3.16; log-rank *p* = 0.043). This endpoint selectivity extends the currently limited literature on *TFAP2D* in lung cancer, but should be interpreted cautiously. Although the association was reproducible across both LUAD and LUSC, it was confined to PFI, with modest effect sizes and relatively wide confidence intervals, and therefore does not support broad claims of prognostic or clinical utility at this stage. One possible interpretation is that *TFAP2D* expression may be more closely related to post-diagnosis disease progression than to mortality-based endpoints such as OS, which can be influenced by additional factors such as subsequent treatment and comorbidity burden. Accordingly, *TFAP2D* should be considered a candidate indicator of disease dynamics requiring further validation. A prior family-level analysis found that selected AP-2 members have prognostic significance in lung carcinoma; however, the contribution of *TFAP2D* remained unclear [45]. More recently, all five AP-2 TFs in LUAD and LUSC were examined, but survival analysis was limited to OS rather than a broader endpoint panel [19]. AP-2 can exert both tumor-promoting and tumor-restraining effects depending on lineage and molecular background. In lung cancer specifically, AP-2α and AP-2β have yielded more direct evidence regarding prognostic or tumor-promoting potential [46,47,48].

### 3.3. TFAP2D Stratification Aligns Primarily with Molecular Classification Variables

**Figure 3:**
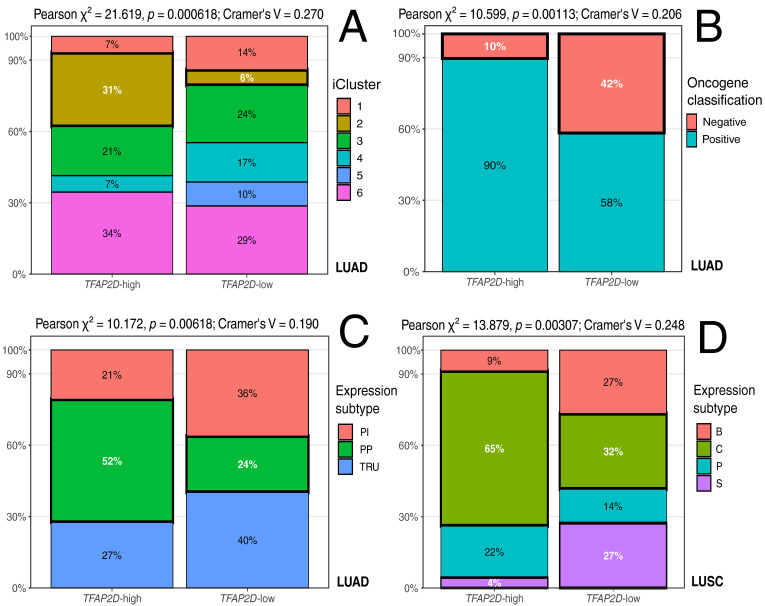
Selected molecular and clinical features of *TFAP2D*-stratified LUAD and LUSC cohorts. (**A**) Distribution of iCluster classes in LUAD. (**B**) Oncogene classification in LUAD. (**C**) Expression subtypes in LUAD. (**D**) Expression subtypes in LUSC. The panels highlight the variables showing the clearest differences between *TFAP2D*-high and *TFAP2D*-low tumors. PI: proximal-inflammatory; PP: proximal-proliferative; TRU: terminal respiratory unit; B: basal; C: classical; P: primitive; S: secretory.

A systematic screen of clinicopathologic and molecular annotations did not identify any broad *TFAP2D*-associated shifts among routine variables in either cohort. Instead, the clearest differences were confined to molecular classification features (Figure 3). In LUAD, *TFAP2D*-high tumors were strongly enriched for iCluster 2 (*p* = 0.000618, post hoc OR = 6.35) and for the oncogene-positive group (*p* = 0.00113, post hoc OR = 6.16). Such patterns place *TFAP2D* expression within a defined molecular background rather than a nonspecific clinicopathologic phenotype. This interpretation is biologically plausible because the TCGA-LUAD framework treats iClusters as integrated molecular states derived from mRNA, methylation, copy-number, and other genomic layers. At the same time, oncogene-based classes capture driver-defined tumor contexts rather than broad histologic variation [49]. Hence, *TFAP2D*-high tumors are more likely to be present in the oncogene-positive fraction; this observation suggests a model in which elevated *TFAP2D* preferentially marks transcriptional states embedded in oncogene-driven LUAD programs rather than a generic clinical subtype. In LUAD, a weaker signal was also observed for expression subtype, driven primarily by enrichment of the proximal-proliferative (PP) subtype in *TFAP2D*-high tumors (*p* = 0.00618; post hoc OR = 3.44). Established LUAD subtyping indicates that transcriptional classes differ in genomic architecture, therapy response, and clinical behavior, with terminal respiratory unit tumors generally linked to EGFR-associated biology and a more favorable prognosis. In contrast, PP tumors exhibit a more proliferation-oriented molecular profile and distinct driver context [49,50]. In LUSC, expression subtype was found to be associated with *TFAP2D*-high tumors (*p* = 0.00307): both those enriched for the classical subtype (post hoc OR = 3.98) and those devoid of the secretory subtype (post hoc OR = 0.12). The canonical LUSC subtype framework distinguishes classical, secretory, basal, and primitive tumors as reproducible transcriptional states with distinct underlying biology [51]. Subsequent immune-landscape analyses indicate that the secretory subtype carries a stronger immune signal, whereas the classical subtype is more closely linked to proliferative and cell cycle-related programs [52]. Hence, the observed *TFAP2D* shift toward classical rather than secretory LUSC is consistent with the cell cycle enrichment observed in the functional analysis of AP-2δ target genes. Full cohort-wide screening results and level-wise post hoc analyses are provided in Supplementary File S1.

### 3.4. Differential Expression Reveals Shared and Histology-Specific TFAP2D-Associated Gene Signatures with Distinct Functional Implications in LUAD and LUSC

**Figure 4:**
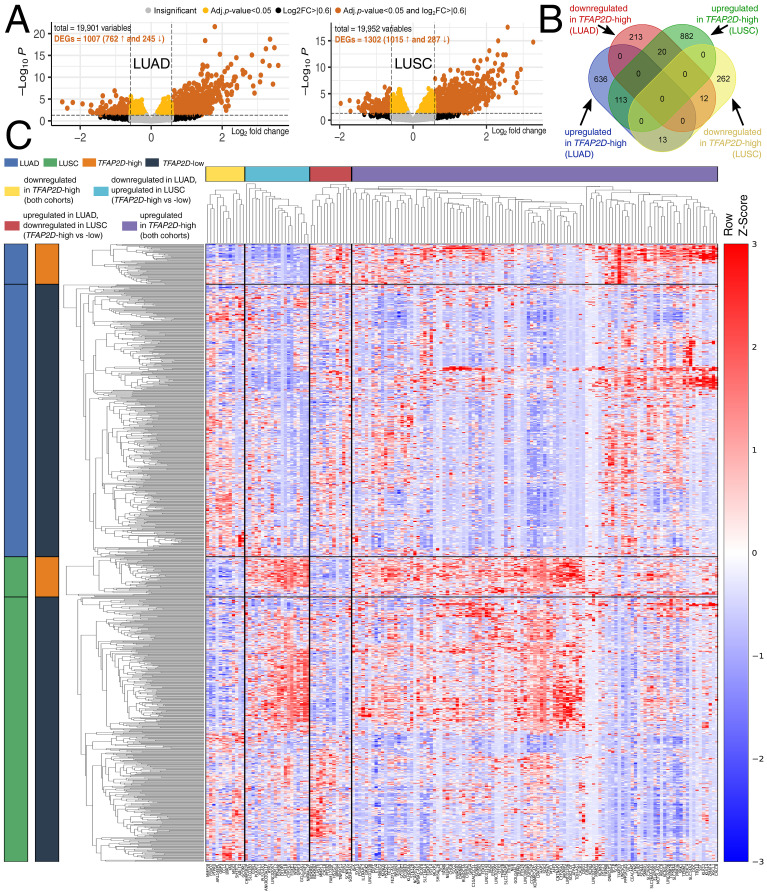
Differential expression and intersection analysis in *TFAP2D*-stratified LUAD and LUSC. (**A**) Differentially expressed genes identified in *TFAP2D*-high vs. *TFAP2D*-low tumors in both tumor cohorts. (**B**) Intersection of the DEA results, showing genes shared between cohorts, as well as genes with cohort-specific or opposite-direction changes. (**C**) Heatmap of intersection-derived gene sets across LUAD and LUSC, showing samples grouped by cohort and *TFAP2D* status, with genes arranged according to the predefined overlap categories.

Using the same *TFAP2D*-high vs. *TFAP2D*-low grouping defined above, differential expression analysis identified 762 upregulated and 245 downregulated genes in *TFAP2D*-high LUAD tumors, as well as 1015 upregulated and 287 downregulated genes in *TFAP2D*-high LUSC tumors (Figure 4A). Other AP-2 family members have also been shown to support oncogenic transcriptional programs in NSCLC, including direct activation of downstream effectors such as USP22 [53], while AP-2β has also been linked to proliferative signaling in NSCLC [47]. Intersection of DEG lists revealed both conserved and histology-dependent components of the AP-2δ–associated transcriptome (Figure 4B). Furthermore, in the *TFAP2D*-high tumors, 113 genes were upregulated in both cohorts, and 12 were downregulated. In addition, 13 genes were upregulated in *TFAP2D*-high LUAD but downregulated in *TFAP2D*-high LUSC, and 20 others showed the opposite configuration. The heatmap of overlap-derived genes confirmed this pattern at the sample level (Figure 4C). Thus, *TFAP2D* stratification identified both a common NSCLC-level regulatory axis and a histology-dependent inversion associated with it. This is consistent with the broader behavior of the AP-2 family, whose members can generate different (or even opposing) transcriptional outputs depending on cellular context and associated regulatory partners [1,6].

This interpretation is supported by the functional annotation of overlap-derived gene sets (Table 1). The genes upregulated in both cohorts (concordantly upregulated) were enriched for inflammatory and cell death-related programs, such as TNF, interleukin-1, NOD-like receptor, and RIPK1/necroptosis terms. In contrast, the concordantly downregulated genes were enriched for neuronal and ion-transport functions, including potassium-channel and neuroactive signaling categories. The discordant sets were separated into xenobiotic/eicosanoid/cytochrome P450 metabolism on one side, as well as calcium- and receptor-linked signaling (IL-17 and NF-κB) on the other. The inflammatory and cell death signaling pathways were also notable: TNF, NOD-like receptor, and RIPK1/necroptosis terms were coenriched, placing part of the shared AP-2δ–associated signature at the interface of innate immune signaling and regulated cell death. In turn, eicosanoid and cytochrome P450 circuits were established components of NSCLC progression and tumor–microenvironment interactions [54,55,56]. IL-17/NF-κB coupling has also been repeatedly implicated in the biology of squamous carcinoma in various epithelial sites [57,58]. Recent LUAD data further support this view by showing that immunogenic cell death-associated transcriptional programs are tightly linked to immune regulation, prognosis, and microenvironmental organization [59]. The neuronal/ion-transport signal should not be dismissed as incidental, because potassium channel dysregulation is increasingly recognized as part of malignant signaling [60]. Together, these enrichments suggest that the difference between LUAD and LUSC reflects the magnitude of the signal associated with *TFAP2D*, as well as the functional orientation of the underlying gene sets. However, these annotations should be interpreted as pathway-level associations rather than direct evidence of phenotypic engagement. The full results of the differential expression analysis, overlap membership, and enrichment outputs underlying Table 1 are provided in Supplementary File S2.

### 3.5. Chromatin Compartment Organization Differed by TFAP2D Stratification, with a Stronger Signal in LUSC than in LUAD

The transcriptome-level analyses presented above defined *TFAP2D*-associated states in LUAD and LUSC, but they did not resolve whether these states extend to higher-order regulatory organization. Therefore, the next two sections examine chromatin compartment organization and cofactor-centered regulatory wiring as complementary routes of investigation. A separate structure-based analysis was then employed to assess whether AP-2δ also presents reproducible candidate pockets for future ligandability-oriented investigation. These follow-up directions were motivated by prior AP-2δ-focused work that highlighted its structural tractability and potential influence on chromatin organization [8]. The first of these complementary analyses was chromatin compartment profiling. Techniques based on 450k methylation data can be used to identify Hi-C-like A/B compartment structure, which broadly corresponds to open and closed chromatin environments [30]. Moreover, three-dimensional chromatin architecture and compartment switching are increasingly recognized as functionally relevant layers of cancer-associated gene regulation rather than passive structural correlates [61,62,63]. This framework is particularly relevant in lung cancer, where LUAD and LUSC exhibit distinct transcriptomic profiles as well as genomic, epigenomic, and lineage-associated regulatory architectures [49,64,65].

**Figure 5:**
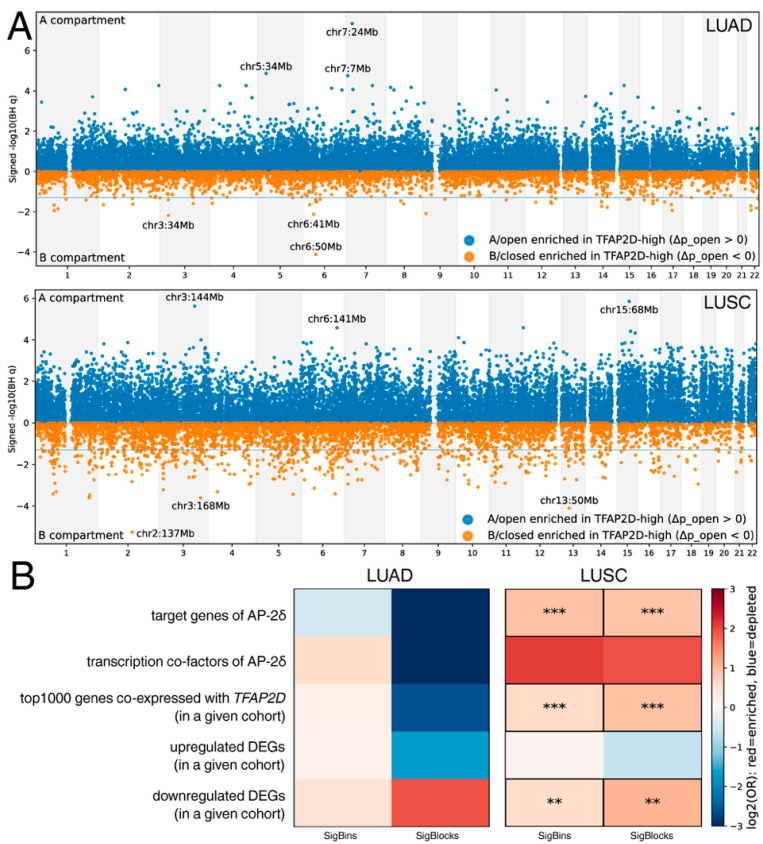
Chromatin compartment analysis in *TFAP2D*-stratified LUAD and LUSC. (**A**) Genome-wide summary of methylation-derived chromatin organization differences between *TFAP2D*-high and *TFAP2D*-low samples in LUAD and LUSC. (**B**) Heatmap summary of enrichment and depletion for selected *TFAP2D*-linked gene sets across significant compartment loci in LUAD and LUSC. SigBins: individual significant bins at adjusted *p*-value < 0.05; SigBlocks: contiguous regions formed by significant bins at adjusted *p*-value < 0.05. ** *p* < 0.01, *** *p* < 0.001.

While chromatin compartment organization was found to differ between *TFAP2D* strata in both cohorts, LUSC exhibited a markedly broader genomic footprint than LUAD (Figure 5A). In LUAD, 1471 of 21,233 bins were significant (FDR < 0.05); of these, 1423 (96.7%) exhibited a higher fraction of A-like assignments in *TFAP2D*-high tumors. Aggregation of significant bins yielded 31 contiguous blocks, with a median length of ~0.3 Mb and a maximum length of ~0.6 Mb. In LUSC, 4211 out of 21,441 bins were significant (FDR < 0.05), with 3701 (87.9%) showing a higher fraction of A-like assignments in *TFAP2D*-high tumors. Block aggregation yielded 219 significant regions, again with a median length of ~0.3 Mb, but extending up to ~0.9 Mb. Importantly, the two cohorts exhibited a similar median shift in A-like assignment among significant bins (LUAD Δp_open = 0.226, IQR = 0.210–0.253; LUSC Δp_open = 0.229, IQR = 0.204–0.265), indicating that the cohorts differed more with regard to genomic extent and regional coherence than per-locus effect size. Compartment changes at individual loci do not need to be very large to be biologically relevant, and even relatively modest fractions of switched bins can accompany meaningful changes in transcriptional organization [63]. In lung cancer, integrated analyses have already linked three-dimensional genome features to expression consequences [66], while lineage-defining transcriptional programs have been shown to shape chromatin state in LUAD [67]. Our findings suggest that while *TFAP2D*-linked differences in chromatin state are detectable in both NSCLC subtypes, they are substantially more extensive and spatially coherent in LUSC.

Afterward, functional overlap analysis assessed whether significant compartment loci preferentially coincided with *TFAP2D*-linked genomic neighborhoods (Figure 5B). In LUSC, the AP-2δ target set showed strong enrichment in both significant bins and merged blocks (SigBins OR = 1.85, FDR = 1.3 × 10^−23^; SigBlocks OR = 1.80, FDR = 1.9 × 10^−6^). In addition, the LUSC top-1000 *TFAP2D* coexpression genes were enriched in both categories (SigBins OR = 1.52, FDR = 1.0 × 10^−5^; SigBlocks OR = 1.87, FDR = 4.0 × 10^−4^), as was the LUSC downregulated DEG set (SigBins OR = 1.44, FDR = 4.2 × 10^−3^; SigBlocks OR = 2.05, FDR = 1.0 × 10^−3^). In contrast, none of the analogous LUAD gene sets reached significance in either the bin-level or block-level analyses, despite showing mixed effect sizes. Full bin-level and block-level outputs are provided in Supplementary File S3. Collectively, *TFAP2D*-high status in LUSC was associated with a broader compartment-level footprint and loci that preferentially coincide with *TFAP2D*-related regulatory neighborhoods; these findings indicate a relatively coherent chromatin-associated pattern. In LUAD, *TFAP2D* stratification is still associated with detectable compartment-level differences, but these appear narrower in scope and less consistently anchored to canonical *TFAP2D*-linked loci. Prior family-level literature supports this interpretation, as AP-2-dependent transcriptional programs are known to be strongly context-dependent and shaped by chromatin engagement rather than by DNA binding alone [1,39].

As chromatin compartments influence transcription through the large-scale partitioning of active and inactive genomic environments, the observed LUSC footprint provides a plausible route by which *TFAP2D*-associated states may couple to broader transcriptional differences. In lung cancer, alterations in three-dimensional genome organization and compartment structure have already been linked to transcriptional consequences and disease-state variation [66,68]. A family-level link is also biologically plausible, as AP-2 paralogs have been shown to shape chromatin accessibility and enhancer engagement in a context-dependent manner [39]. This broader view complies with recent LUAD research showing that tumor progression is closely linked to spatially organized states such as macrophage-associated programs, underscoring that tumor-state identity is shaped by interacting regulatory and microenvironmental layers [69].

### 3.6. TFAP2D-Centered Cofactor Coupling Is More Dynamic in LUAD, and AP-2δ Targets Show Modest Genome-Wide Enrichment

**Figure 6:**
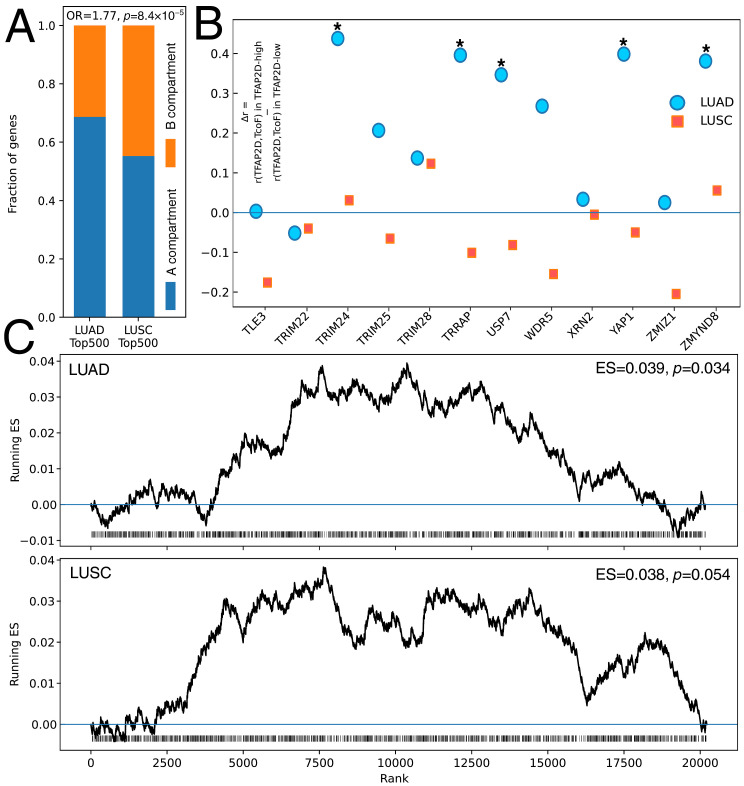
Chromatin-context, cofactor, and target-set enrichment analyses in *TFAP2D*-stratified LUAD and LUSC. (**A**) Distribution of methylation-derived A/B compartment annotations among the top-ranked genes associated with *TFAP2D* in LUAD and LUSC. (**B**) Differential coexpression of the TcoF panel between *TFAP2D*-high and *TFAP2D*-low tumors. (**C**) Genome-wide enrichment of AP-2δ targets within *TFAP2D*-centered gene lists ranked in LUAD and LUSC. *p* < 0.05 (*).

As LUSC was found to have a broader chromatin-compartment footprint than LUAD, the next stage evaluated whether the weaker LUAD signal might be counterbalanced by more localized *TFAP2D*-linked cofactor coupling. Following the chromatin context composition of top-ranked neighborhoods associated with *TFAP2D*, differential coexpression analysis of the TcoF panel and genome-wide enrichment of AP-2δ targets were performed. For the top 500 genes most strongly correlated with *TFAP2D*, the LUSC set showed a significantly different A/B compartment composition to the corresponding LUAD set (Figure 6A), with a relative shift toward B-like compartment assignment in LUSC (OR = 1.77, *p* = 8.4 × 10^−5^). In other words, the genes most closely associated with *TFAP2D* in LUSC appear to partly reside in a different compartment background than the top-ranked LUAD genes, which is consistent with the broader compartment-level analysis in the previous section. Methylation-derived A/B compartments are known to capture large-scale chromatin environments rather than local TF wiring [30], and cancer-associated compartment shifts can coexist with more localized or state-dependent regulatory changes [61,66].

The relationships between *TFAP2D* and TcoFs were tested within the *TFAP2D*-high and *TFAP2D*-low strata (Figure 6B). In LUAD, significant positive rewiring was found in five cofactors: TRIM24 (Δr = 0.447, FDR = 0.0035), TRRAP (Δr = 0.396, FDR = 0.0101), YAP1 (Δr = 0.399, FDR = 0.0101), ZMYND8 (Δr = 0.381, FDR = 0.0109), and USP7 (Δr = 0.346, FDR = 0.0217), whereas WDR5 remained nominal (Δr = 0.268, *p* = 0.0417, FDR = 0.0834). In LUSC, no TcoF reached significance, suggesting that the two tumor cohorts exhibit *TFAP2D*-associated differences at different regulatory levels: LUSC showed a broader, large-scale chromatin footprint, whereas LUAD showed rewiring within a focused cofactor set. The LUAD-associated TcoFs are also biologically credible as regulatory rather than purely descriptive markers. TRRAP is a transcriptional cofactor within SAGA/TIP60-associated assemblies; it has recently been linked to the regulation of MYC- and E2F-target genes while structurally serving as an activator-binding module within the human TIP60 complex [70,71]. ZMYND8 is a chromatin reader associated with transcriptional coregulator networks, NuRD recruitment, and DNA damage-associated transcriptional control [72,73]. YAP1 is a well-established effector of lung adenocarcinoma progression and survival signaling [74,75], USP7 supports NSCLC proliferation and metabolic fitness [76,77], and TRIM24 has been associated with NSCLC aggressiveness and oncogenic transcriptional coactivation in other cancer settings [78,79]. This interpretation is also compatible with recent systems-level evidence showing that lung cancer progression is accompanied by transitions in gene-regulatory network architecture and shifts in central transcriptional regulators [80].

Subsequently, to assess whether AP-2δ targets also show genome-wide enrichment within *TFAP2D*-centered ranked neighborhoods, a preranked enrichment analysis was performed (Figure 6C). In LUAD, enrichment reached significance (ES = 0.039, *p* = 0.034), whereas in LUSC, a similar size effect remained just above the threshold (ES = 0.038, *p* = 0.054). Complete rewiring statistics and genome-wide enrichment outputs are provided in Supplementary File S4. Together, these findings indicate that AP-2δ operates within distinct regulatory architectures in both NSCLC subtypes, with LUSC characterized by broader chromatin-state organization and LUAD by more selective cofactor coupling. These two layers should be viewed as complementary rather than contradictory. In LUAD, the weaker compartment-level signal together with selective rewiring of TRRAP, ZMYND8, YAP1, TRIM24, and USP7 is more consistent with a localized, partner-dependent mode of transcriptional control. In LUSC, the broader and more coherent compartment-level footprint suggests that *TFAP2D*-associated differences are embedded in large-scale chromatin organization, potentially extending across wider regulatory neighborhoods. This interpretation is biologically plausible in light of family-level evidence showing that AP-2 paralogs can act through chromatin-accessibility control and enhancer engagement, whereas partner composition within the conserved dimeric architecture can shape context-dependent transcriptional output [2,39,81]. Nevertheless, this framework should be regarded as exploratory rather than as a definitive model.

### 3.7. Consensus Mapping Prioritized Pockets Within High-Confidence Regions of AP-2δ Structure

**Figure 7:**
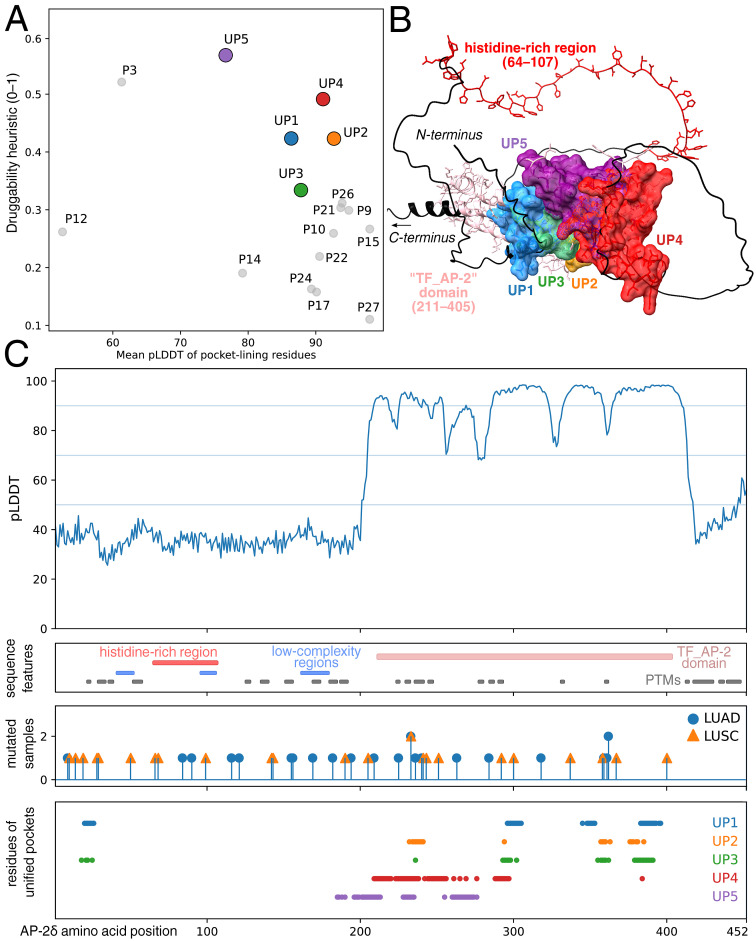
Structure-based pocket investigation of AP-2δ in the context of LUAD and LUSC. (**A**) Cross-tool consensus pocket mapping on the AlphaFold model of AP-2δ, showing the five unified pockets (UP1–UP5) retained after the integration of multiple pocket-detection approaches. (**B**) Three-dimensional localization of the unified pockets, highlighting their concentration within the structured TF_AP-2 domain. (**C**) Residue-level alignment of AP-2δ amino acid positions across four complementary tracks: pLDDT-based model confidence (first), sequence features derived from our previous study [8] (second), mutations identified in LUAD and LUSC (third), and residues assigned to unified pockets (fourth). PTMs: post-translational modifications.

As a final complementary step, the AP-2δ structure was examined for reproducible surface cavities that could be utilized in future ligandability-oriented research. Because no experimental structure of AP-2δ is currently available, this assessment relied on cross-tool consensus mapping applied to the AlphaFold model. Five unified pockets were identified (UP1-UP5), each supported across PrankWeb/P2Rank, FTMap, MOLE 2.5, and DoGSite3 (Figure 7A; Supplementary File S5). Within this shortlist, UP1–UP3 consistently outperformed UP4–UP5 across the main scoring layers. UP1 combined the highest PrankWeb score (5.85), largest PrankWeb pocket volume (868.9 Å^3^), greatest DoGSite3 cavity volume (303.6 Å^3^), and the highest maximum bottleneck free radius detected by MOLE 2.5 (2.711 Å), supporting an accessibility- and geometry-oriented prioritization. UP2 showed the strongest hotspot-centered signal, with the highest FTMap score sum (18,883 vs. 7931 for UP1 and 8602 for UP3) while retaining a high DoGSite3 depth (12.32 Å) and substantial cavity volume (284.2 Å^3^). UP3 remained a credible intermediate candidate, with a PrankWeb score of 5.04, a pocket volume of 636.7 Å^3^, an FTMap score sum of 8602, and a DoGSite3 depth of 10.70 Å. In contrast, UP4 and UP5 were weaker across most axes, with markedly lower PrankWeb scores (1.35 and 1.02), smaller cavity dimensions, and minimal FTMap support (89 and 159, respectively). This type of multi-metric prioritization is particularly relevant for transcription factors, which often do not present one dominant enzyme-like pocket. In such settings, the most credible candidates are those supported consistently across several complementary criteria rather than by a single score [82,83,84].

Three-dimensional mapping indicated that these five unified pockets cluster within the TF_AP-2 domain and are not uniformly distributed across the sequence (Figure 7B,C). This spatial pattern is important because some unified pockets, particularly UP1 and UP3, appear greatly discontinuous when projected onto the linear sequence. Thus, the leading ligandability candidates are concentrated within the family-conserved region responsible for DNA binding and dimerization [2,4]. Importantly, UP1 and UP2 should be viewed as complementary top candidates rather than mutually competing calls: UP1 is favored when accessibility and pocket geometry are weighted most strongly, whereas UP2 is favored when hotspot density is prioritized. This observation was further reinforced by the residue-level confidence, which showed that UP-associated residues were predominantly concentrated within the high-confidence region of the model (Figure 7C). This is particularly relevant because AP-2δ currently lacks an experimental structure; as such, model confidence is an important filter for ligandability-oriented interpretation.

To further contextualize the structural and regulatory layers, sequence features of AP-2δ from our previous study [8] were added (Figure 7C), including the aforementioned TF_AP-2 domain together with the histidine-rich region (HRR), low-complexity regions (LCRs), and predicted post-translational modifications (PTMs). This overlay indicates that cavities do not overlap with the HRR/LCR-containing segment, suggesting that the most plausible direct consequence of ligand engagement at the leading pockets (UP1, UP2) would be the perturbation of AP-2δ functions linked to DNA binding, dimerization, and chromatin engagement. Within this framework, broader chromatin-state behavior in LUSC appears to be the most immediate predicted readout. Effects on LUAD-associated cofactor rewiring would more likely be indirect and context-dependent, although the distributed pattern of predicted PTM-bearing segments does not exclude broader downstream regulatory consequences. The mutation overlay has provided LUAD/LUSC-specific context but not an independent prioritization signal (Figure 7C). Overlaying variant positions with UP residue tracks enabled the direct assessment of whether any leading pocket coincided with mutation-enriched regions, but the available pattern remained sparse, and no strongly recurrent hotspot was concentrated within the top UPs.

Taken together, the present study proposed a small, quantitatively prioritized set of reproducible AP-2δ cavities that should be interpreted as structurally plausible candidates rather than validated ligand-binding sites, with their main value lying in the identification of regions for future experimental investigation. This is consistent with the broader shift from viewing transcription factors as categorically inaccessible to recognizing that selected protein–DNA and protein–protein interfaces can provide tractable entry points for chemical biology [82,83,84]. Structural information for AP-2δ remains limited, so the present analysis should be viewed primarily as a resource for future mechanistic and chemical biology studies.

Some limitations of the present study should be acknowledged. It was a computational analysis of publicly available cohorts that integrated transcriptomic data, clinical annotations, and methylation-based inference of chromatin compartment state. Accordingly, the reported associations should not be interpreted as causal and may still be influenced by cohort composition, annotation structure, and constraints imposed by available multi-omic data. *TFAP2D* stratification was expression-based and therefore should be regarded as a simplification of what is likely to be a continuous, context-dependent, and potentially non-linear regulatory signal. The survival signal associated with *TFAP2D* was restricted to PFI, with modest hazard ratios and relatively wide confidence intervals, limiting immediate prognostic interpretation and underscoring the need for independent validation. The chromatin layer relied on methylation-derived compartment proxies rather than matched Hi-C or Micro-C profiling, meaning that large-scale chromatin context could be inferred but not directly measured at high structural resolution. Likewise, the cofactor analyses captured state-dependent association and rewiring within the TcoF panel but did not establish a regulatory hierarchy. The structural section should also be interpreted within an appropriate scope: candidate ligandable surfaces were prioritized using an AlphaFold-guided consensus framework and cross-tool triangulation, but no chemical or biophysical validation was performed. Therefore, their present value lies in proposing regions for future experimental testing, not in establishing confirmed ligandability. Overall, no genetic or pharmacological perturbation was performed, and no orthogonal validation of *TFAP2D* expression or activity was available. The reported patterns should be interpreted as state-linked associations rather than functional dependencies. Likewise, enrichment of inflammatory, cell-death, or chromatin-associated programs did not establish involvement at the phenotypic level.

However, these limitations should be considered alongside the study’s main strengths. This research provides the first *TFAP2D*-focused analysis of lung cancer, integrating clinical outcome, molecular subclassification, cross-cohort transcriptomic structure, shared vs. divergent DEG programs, compartment-level chromatin context, cofactor rewiring, and structure-guided pocket prioritization within a single LUAD/LUSC framework. Also, the study establishes a coherent, testable map of *TFAP2D*-associated biology across multiple regulatory layers. It is particularly relevant given the limited prior *TFAP2D* literature in lung cancer. Another strength is that the observed LUAD-LUSC differences were not collapsed into a single pooled interpretation but instead resolved into distinct regulatory ranges: LUSC revealed a broader chromatin-compartment footprint, whereas LUAD showed more pronounced selective cofactor rewiring. This distinction makes the biological model more informative and also more experimentally actionable.

From a clinical perspective, the current relevance of *TFAP2D* remains exploratory: stratification aligned more strongly with molecular subclassification than with broad clinical variables, suggesting that its main value may lie in identifying biologically distinct tumor states rather than in routine classification. Moreover, the association with PFI and the observed subtype-dependent regulatory architecture support *TFAP2D* as a candidate indicator of progression-linked disease dynamics and as a potential entry point for therapeutic investigation across tumor subtypes. Future assessment of clinical applicability requires prospective validation, standardized cutoffs, and the inclusion of variables beyond those currently used. Other directions for further research include: *TFAP2D*/AP-2δ perturbation coupled with RNA-Seq/ATAC-Seq/Hi-C/Micro-C; expansion of the cofactor panel beyond the current set; and experimental assessment of the prioritized pockets through docking, fragment screening, and structural biology. Taken together, the present study positions AP-2δ as an underexplored but mechanistically tractable candidate whose role in lung cancer appears biologically meaningful and non-uniform across histologies, with sufficient support to justify experimental follow-up.

## 4. Conclusions

This study provides an integrated characterization of *TFAP2D*/AP-2δ in lung cancer by combining transcriptomic, molecular classification, chromatin context, cofactor network, and structure-guided analyses across LUAD and LUSC. *TFAP2D* stratification delineated biologically distinct states in both histologies, but the associated regulatory architectures were not identical. *TFAP2D* was linked to modular gene expression structure and molecular subclassification, as well as shared and histology-dependent differential expression programs. Clinical profiling further suggested that *TFAP2D* stratification was more strongly associated with molecular subtype composition than with broad clinicopathological differences. The consistent survival association was observed for progression-free interval, with broader survival effects absent across endpoints. LUSC exhibited a broader and more coherent chromatin compartment footprint, whereas LUAD showed more pronounced selective rewiring within the *TFAP2D*-centered cofactor network. Structural prioritization further identified a small set of reproducible candidate pockets, with the strongest candidates concentrated within the ordered TF_AP-2 domain and supported by either accessibility- and geometry-focused features or by hotspot- and energetics-oriented characteristics. Taken together, these findings suggest that AP-2δ is a component of distinct regulatory architectures in LUAD and LUSC. This work therefore provides a practical framework for future studies by prioritizing mechanistic validation of *TFAP2D*-dependent regulatory programs, direct testing of its relationship with chromatin organization and cofactor coupling, and structure-guided exploration of the high-confidence ligandability candidates. The present study positions AP-2δ as an underexplored but biologically relevant candidate for further research aimed at defining histology-specific regulatory vulnerabilities in lung cancer.

## Figures and Tables

**Table 1:** Functional enrichment of *TFAP2D*-associated gene sets from intersection analysis.

Gene Set	Ontological Category	Functional Annotation	*p*-Value
Downregulated in *TFAP2D*-high vs. -low (both cohorts)	GO:BP	Regulation of apoptotic process	9.22 × 10^−3^
		Negative regulation of cell population proliferation	2.11 × 10^−2^
		Cellular response to interleukin-1	2.83 × 10^−2^
	KEGG	TNF signaling pathway	1.52 × 10^−3^
		Herpes simplex virus 1 infection	3.56 × 10^−3^
		NOD-like receptor signaling pathway	3.79 × 10^−3^
	Reactome	RIPK1-mediated regulated necrosis	1.60 × 10^−2^
		Regulation of necroptotic cell death	1.60 × 10^−2^
		Regulated necrosis	3.02 × 10^−2^
Downregulated in LUAD, upregulated in LUSC (*TFAP2D*-high vs. -low)	GO:BP	Xenobiotic metabolic process	2.98 × 10^−3^
		Icosanoid metabolic process	7.97 × 10^−3^
		Epoxygenase P450 pathway	1.32 × 10^−2^
	KEGG	N.A.	N.A.
		N.A.	N.A.
		N.A.	N.A.
	Reactome	Miscellaneous substrates	8.96 × 10^−3^
		Metabolism of lipids	1.23 × 10^−2^
		Cytochrome P450—arranged by substrate type	4.61 × 10^−2^
Upregulated in LUAD, downregulated in LUSC (*TFAP2D*-high vs. -low)	GO:BP	Positive regulation of cytosolic calcium ion concentration	1.76 × 10^−3^
		Negative regulation of intrinsic apoptotic signaling pathway	2.30 × 10^−3^
		Mammary gland alveolus development	7.82 × 10^−3^
	KEGG	Pathways in cancer	5.73 × 10^−4^
		IL-17 signaling pathway	2.67 × 10^−3^
		NF-kappa B signaling pathway	3.25 × 10^−3^
	Reactome	Peptide ligand-binding receptors	1.03 × 10^−2^
		G alpha (i) signaling events	2.47 × 10^−2^
		Class A/1 (Rhodopsin-like receptors)	2.73 × 10^−2^
Upregulated in *TFAP2D*-high vs. -low (both cohorts)	GO:BP	Monoatomic ion transmembrane transport	1.31 × 10^−7^
		Regulation of presynaptic membrane potential	7.96 × 10^−6^
		Potassium ion import across plasma membrane	3.94 × 10^−5^
	KEGG	Glutamatergic synapse	1.18 × 10^−2^
		Cholinergic synapse	1.18 × 10^−2^
		Neuroactive ligand-receptor interaction	1.69 × 10^−2^
	Reactome	Neuronal system	4.32 × 10^−6^
		Potassium channels	1.35 × 10^−4^
		Inwardly rectifying K+ channels	6.30 × 10^−4^

N.A. indicates that no significantly enriched pathways were identified for the specified ontological category in a given gene set.

## Data Availability

The original contributions presented in this study are included in the article/Supplementary Materials. Further inquiries can be directed to the corresponding author.

## Supplementary Materials

The following supporting information can be downloaded at: https://www.mdpi.com/article/10.3390/cancers18081278/s1. Supplementary File S1. Clinical and molecular profiling of *TFAP2D*-stratified LUAD and LUSC cohorts (Supplementary_File_S1.xlsx). Supplementary File S2. Differential-expression and overlap analyses in *TFAP2D*-stratified LUAD and LUSC cohorts (Supplementary_File_S2.xlsx). Supplementary File S3. Chromatin-compartment analyses in *TFAP2D*-stratified LUAD and LUSC cohorts (Supplementary_File_S3.xlsx). Supplementary File S4. Cofactor rewiring and ranked-neighborhood analyses in *TFAP2D*-stratified LUAD and LUSC cohorts (Supplementary_File_S4.xlsx). Supplementary File S5. Structure-based pocket prioritization of AP-2δ (Supplementary_File_S5.xlsx).
